# Unusual Course of a Pulmonary Artery Catheter Due to Absence of Right Superior Vena Cava

**DOI:** 10.5152/TJAR.2023.22169

**Published:** 2023-04-01

**Authors:** Brendon J Burke, Charl J De Wet, Mohammad A Helwani

**Affiliations:** 1 Department of Anaesthesiology, Washington University, St. Louis, USA

**Keywords:** Absence of right superior vena cava, cardiovascular and thoracic anaesthesia, persistent left superior vena cava, unusual course of a pulmonary artery catheter, vascular access difficulty

## Abstract

The persistent left superior vena cava may complicate the placement of vascular access. It rarely occurs with an absence of the right superior vena cava. We present a chest X-ray of a patient with this rare anomaly that was demonstrated incidentally with an unusual course of a pulmonary artery catheter course.

**Main points**

The persistent left superior vena cava (PLSVC) is the most common congenital venous anomaly of the thoracic venous return.
PLSVC is rarely associated with the absence of the right superior vena cava. - Mostly detected incidentally on chest imaging and suspected with dilated CS on echocardiography.
PLSVC may complicate vascular access and cardiac procedures. - Proceduralists should be familiar with this anomaly to avoid complications.

## Introduction

The persistent left superior vena cava (PLSVC) with the absence of the right superior vena cava (RSVC) is a rare venous anomaly. We present a patient with this rare anomaly demonstrated incidentally on a Chest X-ray (CXR) with an unusual course of a pulmonary artery (PA) catheter.

## Case Presentation

A 55-year-old male underwent an emergent aortic root and ascending aorta replacement for type A aortic dissection. A PA catheter was floated in the operating room without difficulty. Intraoperative transoesophageal echocardiography revealed a dilated coronary sinus (CS) and the presence of PLSVC between the left atrium appendage and the left superior pulmonary vein. 

**Figure 1.**
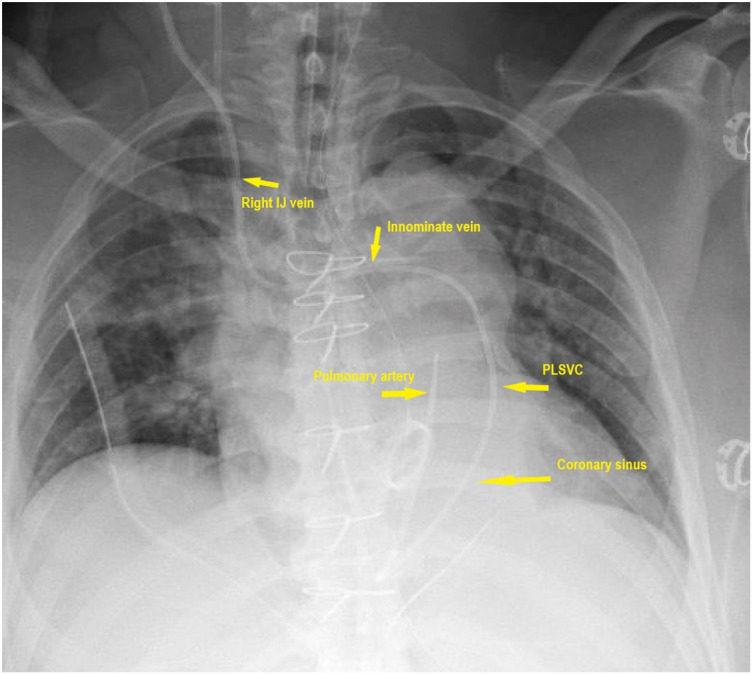
Postoperative CXR showing the unusual course of the PA catheter due to the presence of persistent left and absent right superior vena cava.

A postoperative chest x-ray showed the PA catheter coursing from the right intrajugular vein to the innominate vein making a left due to the absence of RSVC and entering the PLSVC and then into the CS and the right atrium (RA) finishing its course in the PA as shown on the CXR (where the tracing on the PA catheter shows a PA pressure tracing) (Figure 1). 

## Discussion

The PLSVC is a congenital anomaly with a 0.5%-2% incidence mostly diagnosed incidentally. It occurs generally in conjunction with a RSVC with or without a connecting innominate vein. The PLSVC drains to the RA via the CS but can drain into the left atrium via unroofed CS. Only about 10% of PLSVC cases have absent RSVC, as demonstrated in our patient.^1^

The PLSVC is usually detected incidentally on chest imaging and suspected with dilated CS on echocardiography. The diagnosis is confirmed as an agitated saline/contrast study in the left upper extremity as it shows bubbles in the CS before entering the RA on echocardiography.^2^

The PLSVC may complicate the placement of vascular access, PA Catheter (PAC), and transvenous pacemaker including difficulty in insertion and possible vascular injury. It also presents unique difficulties in administering retrograde cardioplegia and during heart transplantation. In patients with PLSVC and unroofed CS, significant right to left shunt may be present.^3^

## Conclusion

The PLSVC is a congenital venous anomaly that may complicate procedures such as vascular access and transvenous pacemaker. It is rarely associated with the absence of the RSVC. Proceduralists need to be familiar with this anomaly to avoid complications.
